# Observing Personality in Everyday Life: How Personality Influences Daily Activities and Mood

**DOI:** 10.1093/geroni/igab046.960

**Published:** 2021-12-17

**Authors:** Shiyang Zhang

## Abstract

Personality reflects the influence of older adults’ past experiences on their tendencies to engage in certain behaviors and generate emotions. An increasing number of studies have focused on the expression of personality in older adults’ everyday life. Specifically, personality features have been associated with daily activities, affect reactivity, and mood. This symposium draws on Ecological Momentary Assessments and longitudinal analysis to understand how personality is manifested in daily life, and how daily emotional experiences accumulate over time to influence physical health. Lee and colleagues examine how personality is associated with daily activities and find that extraversion is positively associated with activity diversity in two adult samples. Pasquini and colleagues consider the within-person fluctuations in personality traits and demonstrate the possibility of using daily behaviors and affect as markers of extraversion and neuroticism. Zhang and Fingerman assess how positive and negative moods concurrently change in reaction to daily social contacts and confirm that narcissism moderates such associations. Finally, this symposium also focuses on the cumulative effect of daily emotions on physical health. Leger and colleagues address the long-term association between personality (e.g., neuroticism, conscientiousness) and physical health and identify the mediating role of negative reactivity to daily stressors. Collectively, the presentations provide an in-depth analysis of personality’s impact on concrete daily behaviors and emotions, as well as their profound long-term effect on physical health. Our discussion outlines future research directions and highlights how inter-individual differences, simultaneous life events, and social interactions intertwine to influence individuals’ behaviors that occur in natural settings.

